# Hybrid Device Fabrication Using Roll-to-Roll Printing for Personal Environmental Monitoring

**DOI:** 10.3390/polym15122687

**Published:** 2023-06-15

**Authors:** Thanh Huy Phung, Anton Nailevich Gafurov, Inyoung Kim, Sung Yong Kim, Kyoung Min Kim, Taik-Min Lee

**Affiliations:** 1 Department of Mechatronics, Ho Chi Minh City University of Technology (HCMUT), 268 Ly Thuong Kiet Street, District 10, Ho Chi Minh City 70000, Vietnam; huypt@hcmut.edu.vn; 2 Vietnam National University Ho Chi Minh City, Linh Trung Ward, Thu Duc, Ho Chi Minh City 70000, Vietnam; 3 Department of Flexible and Printed Electronics, Korea Institute of Machinery and Materials (KIMM), 156 Gajeongbuk-ro, Yuseong-gu, Daejeon 34103, Republic of Korea; anton@kimm.re.kr; 4 Department of Nanomechatronics, Korea University of Science and Technology (UST), 217 Gajeong-ro, Yuseong-gu, Daejeon 34113, Republic of Korea; 5 Department of Robot and Manufacturing System, Korea University of Science and Technology (UST), 217 Gajeong-ro, Yuseong-gu, Daejeon 34113, Republic of Korea; 6 Department of Advanced Materials Engineering, Tech University of Korea (TU Korea), 237 Sangidaehak-ro, Siheung-si 15073, Gyeonggi, Republic of Korea; syongkim@tukorea.ac.kr (S.Y.K.); kkm386@tukorea.ac.kr (K.M.K.)

**Keywords:** roll-to-roll, printed electronics, hybrid electronics, IoT devices, environmental monitoring, screen printing

## Abstract

Roll-to-roll (R2R) printing methods are well known as additive, cost-effective, and ecologically friendly mass-production methods for processing functional materials and fabricating devices. However, implementing R2R printing to fabricate sophisticated devices is challenging because of the efficiency of material processing, the alignment, and the vulnerability of the polymeric substrate during printing. Therefore, this study proposes the fabrication process of a hybrid device to solve the problems. The device was created so that four layers, composed of polymer insulating layers and conductive circuit layers, are entirely screen-printed layer by layer onto a roll of polyethylene terephthalate (PET) film to produce the circuit. Registration control methods were presented to deal with the PET substrate during printing, and then solid-state components and sensors were assembled and soldered to the printed circuits of the completed devices. In this way, the quality of the devices could be ensured, and the devices could be massively used for specific purposes. Specifically, a hybrid device for personal environmental monitoring was fabricated in this study. The importance of environmental challenges to human welfare and sustainable development is growing. As a result, environmental monitoring is essential to protect public health and serve as a basis for policymaking. In addition to the fabrication of the monitoring devices, a whole monitoring system was also developed to collect and process the data. Here, the monitored data from the fabricated device were personally collected via a mobile phone and uploaded to a cloud server for additional processing. The information could then be utilized for local or global monitoring purposes, moving one step toward creating tools for big data analysis and forecasting. The successful deployment of this system could be a foundation for creating and developing systems for other prospective uses.

## 1. Introduction

Along with the connection of the environment to the existence of people and other creatures on Earth, environmental challenges are crucial components of sustainable development together with economic growth. For example, the health of every individual is directly threatened by air pollution from socioeconomic activities, such as manufacturing and transportation [1,2]. Therefore, environmental monitoring is crucial to protect personal health while serving as a foundation for policymaking. The advantages of the Internet of Things (IoT) and big data can now be availed for environmental monitoring in the Fourth Industrial Revolution [3,4,5,6], primarily by personalizing environmental parameter monitoring. Monitoring devices should be mass-produced for this purpose at low costs and in a short cycle time. For such applications, environmentally acceptable green fabrication techniques must also be considered.

Printed electronics have shown great potential for future electronics [7,8]. Specifically, printing techniques are currently evolving toward conformable, elastic, wearable, or skin-like devices [9,10,11,12,13], with complex systems incorporating IoT [14,15,16,17,18,19,20]. The devices and components are directly fabricated in printed electronics through additive processes without intricate and hazardous procedures compared to other fabrication techniques such as photolithography, etching, or electroplating. As a result, cost savings and environmental benefits could be achieved. Roll-to-roll (R2R) printing techniques have been regarded as effective ways to produce printed electronics with low costs, high throughputs, and minimal waste [21,22,23,24,25,26,27,28], with a variety of applications [22,29,30,31,32,33,34].

This study proposes a fabrication method of hybrid printed electronic devices to personalize environmental monitoring. So far, passive and active electrical components can be printed due to advances in printing technologies [35]. However, more steps and printing methods must be integrated to achieve high-quality and stable fully printed devices. Moreover, electronic components and devices fabricated with conventional methods are still popular in the market share due to their long history. As a result, hybrid printed electronics devices (i.e., devices that combine printed circuits and conventional components) have become increasingly popular in recent years [36,37,38,39,40,41]. Note that by using this hybrid approach, the individual electronic components and sensors made by other printing processes can also be incorporated into pre-printed circuits [42].

In this study, the proposed fabrication method was used to develop a hybrid device for personal environmental monitoring. Accordingly, printed circuit boards were fabricated using an R2R screen-printing method. For this purpose, design rules were proposed to ensure the printability and performance of the fabricated devices. Passive and active compensations were applied to align and control the registration during printing. After the multilayer circuit was printed, the solid-state components, such as the microcontroller unit (MCU), Bluetooth module, and sensors, were assembled to achieve complete devices. Metal oxide-based gas sensors [43] were used for air quality monitoring. In addition, a monitoring system prototype utilizing hybrid manufactured devices and an IoT ecosystem, including a mobile application and a cloud server, was developed to customize environmental monitoring further.

## 2. Personal Environmental Monitoring System

**Figure 1:**
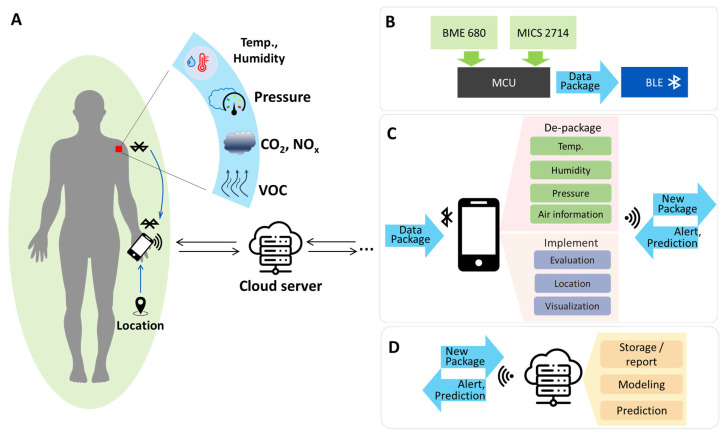
Development of the structure and features of the monitoring system. (**A**) Monitoring system concept, (**B**) monitoring device components, (**C**) mobile application features, and (**D**) cloud server features.

The structure and operation of the personal monitoring system are described in Figure 1. This system senses environmental information using hybrid printed devices (Figure 1A). So-called hybrid printed devices comprise an R2R-printed platform integrated with solid-state components and commercially available sensors.

As illustrated in Figure 1B, the devices acquire the data via environmental sensors, preprocess and bundle the data with the MCU, and transmit the data packages to personal mobile phones via Bluetooth modules within a specified period. After receiving the data packages, the mobile devices analyze them, determine the monitoring values and location, and then display the information in the application (Figure 1C). After a regular interval of time, the information is uploaded to a cloud server (Figure 1D). As shown in Figure 1D, the server collects data from multiple devices and processes them further with data tools to provide information that could be used for environmental status warnings or forecasts. Environmental data and the locations of several prototype monitoring devices were gathered, but personal identifiers were omitted from this study’s prototype system because of privacy concerns.

## 3. Fabrication of Hybrid R2R Printed Device

**Figure 2:**
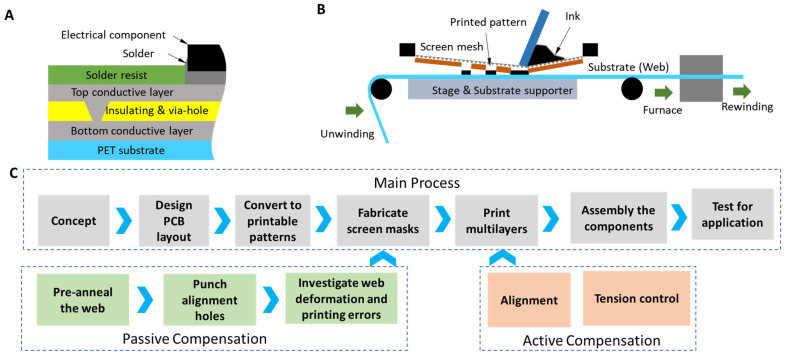
The fabrication method and process for the hybrid devices. (**A**) Hybrid multilayer printed circuit board (PCB) structure, (**B**) roll-to-roll (R2R) screen printing of the circuits, and (**C**) the printing process using R2R printing.

This section describes the fabrication process for the devices examined in this study. In this fabrication process, the device is manufactured by printing the multilayer circuits directly onto the flexible substrate using R2R screen printing, followed by mounting the electrical components and sensors (Figure 2).

Figure 2A illustrates a typical multilayer circuit structure. The circuits comprise four layers: a conductive layer (bottom conductive layer), an insulating layer, another conductive layer (top conductive layer), and a solder resist layer on top. To produce these four layers, the functional inks (such as conductive, insulating, and solder-resist inks) are printed directly onto the flexible substrate to generate these four layers (Figure 2B). Here, the flexible substrate is consistently fed from the unwinding roll to the screen-printing station for printing. After printing, the pattern is transferred to the furnace for sintering or annealing to enhance its functional properties, then forwarded to the winding roll. The four layers are sequentially printed without using complex or hazardous procedures to fabricate the circuits through this fabrication process. This approach could significantly reduce production time, cost, and material waste as well as safeguard the environment and labor health.

However, during the fabrication process using direct R2R printing, the printability, functional performance, and reliability of the fabricated devices must be carefully considered. For this purpose, the fabrication process is proposed (Figure 2C).

First, the printed circuit board (PCB) layout and electronic components are designed and preselected based on the device’s concept (Section 2). This phase determines the circuit wiring and component footprints to be printed without defects or electrical shorts. Note that the difficulty increases because of the substrate’s flexibility.

Second, the design is converted into multiple printable patterns for printing. The converted patterns are then printed layer by layer to develop circuits. However, the substrate is flexible and deformable, so the alignment of the layers may be compromised, resulting in the failure of the final devices. Consequently, compensating for the design during conversion and printing is essential. This study proposes using active and passive compensation for registration control (alignment of the printed layers) to guarantee the performance of the devices.

The components are mounted to the circuit in the following step, which is essential because it determines the performance and reliability of the final devices. After the components are assembled to form a complete device, the device is tested for its intended purpose. The essential details of the processing steps are illustrated in the following sections.

### 3.1. PCB Layer Design

At first, the schematic and the PCB layout were designed based on the fabrication concept. The design must ensure the printed patterns’ printability and performance considering the printing method’s characteristics [44]. For instance, the line width and spacing of the printed patterns must be sufficiently conductive and screen-printable without electrical faults. When the insulating layer is printed, the openings (via holes) must ensure that the top and bottom conductive layers are interconnected. Moreover, the electronic components should be chosen so that their pads can be printed and the devices can be soldered easily. The design rules in Table 1 were proposed for these purposes.

Accordingly, the width of the design patterns for printing should be at least 200 μm, although fine patterns of around 100 μm could also be printed [44]. Surface-mount technology (SMT) devices having the size codes of 1608 (metric, i.e., devices with a dimension of 1.6 mm × 0.8 mm) or larger are recommended considering the later soldering process. Moreover, in this fabrication method, the via holes were directly formed during the printing of the insulating layer instead of drilling and plating, as in the conventional PCB fabrication processes. The diameter of the printed holes should be at least 400 μm, and later, the printed patterns on the top layer should be large enough to cover the holes and create interconnections between the conductive layers (bottom and top layers). In addition, using zero-interconnection-force (ZIF) connectors is convenient because they can be printed directly and minimize external effects that can damage the flexible device. Note that printability, interpreted through the design rules in Table 1, is also the criterion for selecting solid-state components, such as sensors, for the fabricated devices.

### 3.2. Conversion to Printable Patterns and Passive Compensation

After the design process, the layout is converted into four screen meshes to print four layers, which need to be aligned well to make the device. However, during R2R printing, the polymeric web substrate is subjected to high temperatures to anneal the printed layers while bearing tension. As a result, the substrate and printed patterns deform after printing. Moreover, the screen-printing process contributes to positioning errors in the printed patterns. To reduce the influence of such deformations and errors, passive compensation was performed for the design and the web substrate prior to printing.

To passively compensate for the patterns, the experiments on the deformation of the substrate were conducted with the actual printing condition. Here, the web substrate, polyethylene terephthalate (PET; XG7AH7, Toray Industries, Japan), with a thickness of 125 μm and a width of 70 mm, was pre-annealed during a free run at 12 mm/s through 10 m of the 150 °C heating chamber with a web tension of 3 kgf.

**Figure 3:**
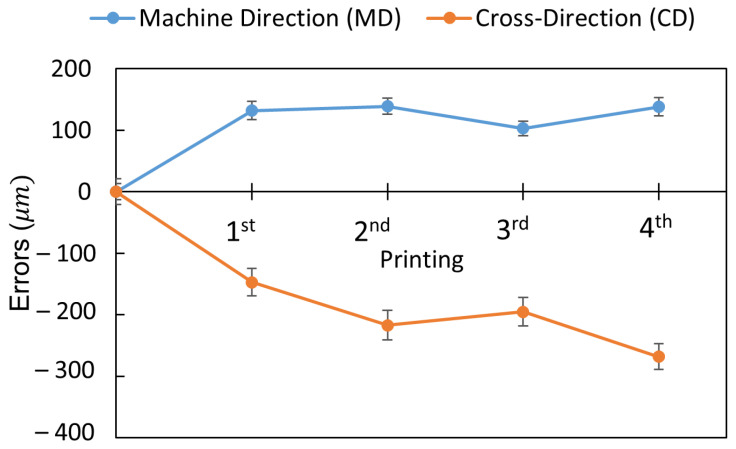
Deformation of the web substrate after each print.

Figure 3 shows the experimental results on the deformation of the pre-annealed PET substrate (70 mm wide and 125 μm thick) with a sintering temperature of 150 °C through four prints. As shown in the figure, the web substrate tends to elongate along the machine direction (MD—along the web-moving direction) and shrink along the cross direction (CD—across the web). Therefore, the dimensions of the layers in the original design need to be scaled for accurate printing. The scale factors used in this study are calculated in ratios and shown in Table 2. Note that the factors should be different according to the substrate materials and printing conditions.

### 3.3. Printing of Multilayer Circuit, Active Compensation, and Component Assembling

**Figure 4:**
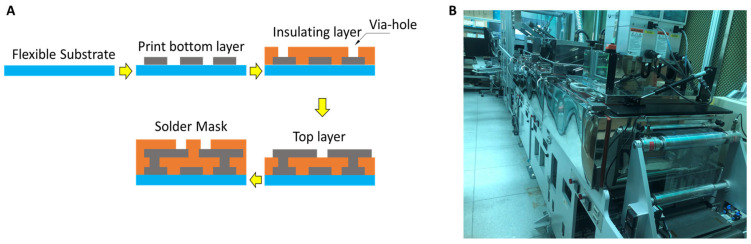
Multilayer circuit printing. (**A**) Multilayer printing, (**B**) the R2R screen printer used for experiments.

After converting the design into the screen meshes and passive compensation, the circuits are printed and sintered through R2R screen printing and assembled with the electronics components. Figure 4 shows the printing sequence of the layers (Figure 4A) and the R2R printer used in this study (Figure 4B).

Table 3 describes the details of the specific materials and meshes used to print the devices. The first and third layers (bottom and top) were printed with silver paste (T40, FP Co., Ltd., Busan, Republic of Korea), while the second and fourth layers were printed with insulating ink (SFR-300 PYI, Seoul Chemical Research Laboratory Co., Ltd., Gyeonggi, Republic of Korea). Note that after printing, the materials usually need to be post-processed to achieve functional properties. In our case, the silver paste should be sintered to be conductive, and the insulating ink should be annealed to create solid layers. Since the substrate used to print the circuit is a vulnerable polymer, the materials should be selected so that the printability and performance of the printed devices can be ensured with low processing temperatures. The fine mesh was used for the conductive layer under the insulating layer to reduce the surface roughness, and a lower mesh-count mesh was used to print an efficient insulating layer. This study utilized a 400 mesh-count screen mask for the bottom layer before printing the insulating layer with a 200 mesh-count screen mask, followed by the top conductive layer and the solder resist layer (325 mesh-count for both).

**Figure 5:**
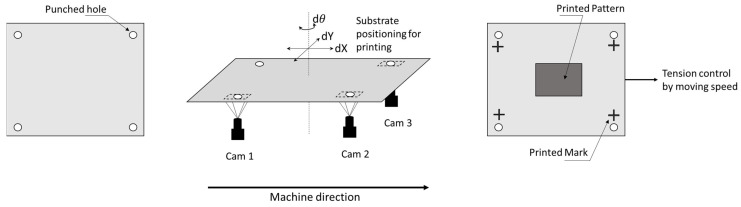
Alignment and active compensation process for multilayer printing.

During printing, the printer uses an alignment system to align the substrate to the screen mask and actively compensates for the registration errors (Figure 5). For alignment and registration control, the pre-annealed substrate is punched before printing, and the holes are used as reference marks. Accordingly, the alignment system uses the cameras to recognize the marks (punched holes) and adjust the relative position of the pattern on the screen masks and the substrate. Moreover, even though passive compensation is applied, misalignment could still occur. Therefore, the layer alignment marks are printed along with the pattern to solve the issue. Then, the cameras compare the positioning errors between the reference marks (holes) and the printed marks, and the system controls the tension of the web substrate via the motion of the web to compensate for errors (active compensation or active registration control) [44,45].

After printing, soldering the components to the printed circuit is a critical step for the operation of the devices [46]. This study used PET as the substrate; therefore, a low-temperature reflowable solder with a melting point of around 150 °C was used to assemble the electrical components. The printable solder paste was put at the printed footprint of the component on the substrate. The components then were attached and annealed for roughly 2 min. The solder paste melted and reflowed under the influence of the solder resist layer and locate the components. During cooling, the components were fixed to the circuit, and the interconnections were made.

## 4. Results and Discussion

### 4.1. Device Fabrication Results

**Figure 6:**
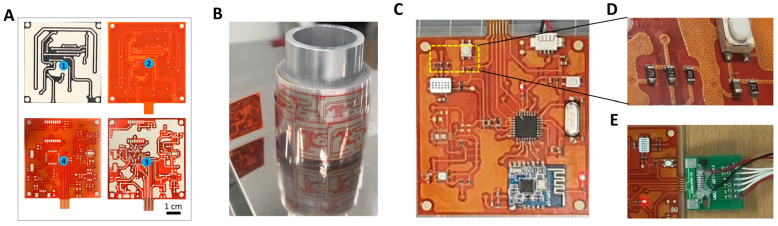
Roll-to-roll printed circuit boards and fabricated monitoring devices. (**A**) Four printed layers of the circuit boards in sequence: ➀ bottom conductive layer, ➁ insulating layer with printed via holes, ➂ top conductive layer, and ➃ solder-resist layer; (**B**) printed circuits after R2R printing; (**C**) fully fabricated device with zero-interconnection-force (ZIF) and non-ZIF connections; (**D**) soldered electrical components; and (**E**) layout of the board while programming the MCU via an external board.

The results of the fabricated devices are shown in Figure 6. Figure 6A shows the four printed layers in sequence on a web roll (Figure 6B). After the circuits were printed, the components were mounted to achieve the completed devices (Figure 6C,D). The soldered connection showed sufficient quality (Figure 6D).

In this study, both ZIF and non-ZIF for the three-level connections (connections made with other external devices [47]) were used in the device. The non-ZIF connector was used for less changeable connections, such as power, while the ZIF connector was more versatile for data transmission, powering, and programming the MCU, as shown in Figure 6E. The fabricated devices showed a working performance almost similar to that of the conventional devices.

**Figure 7:**
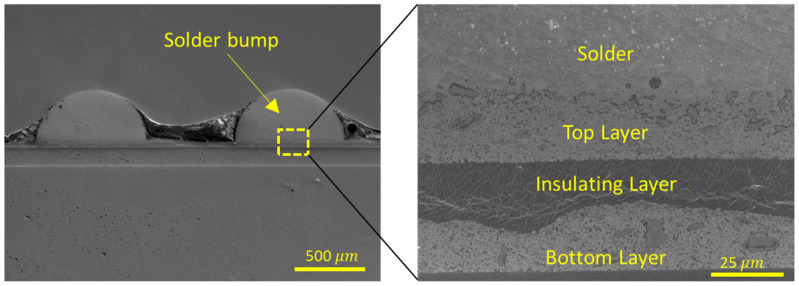
Microstructure of the fabricated device.

Figure 7 shows the microstructure of the printed circuit. The thickness of the printed layers was about 15–20 μm. According to the microstructure, the connections between the layers, between the solder bump and the top, were constructed well without any substrate damage.

### 4.2. Implementation of Personal Environment Monitoring System with Fabricated Device

After fabrication, the device was used with a mobile application and a remote cloud server. With this approach, the device collects data from the sensors and sends them to the mobile application which extracts and uploads the data to the server. The server uses an external library to estimate more information, send it back to mobile phones, and execute additional tasks.

The sensors collect and process the data in the monitoring device and sent them to the MCU. Here, the characteristic of the NOx sensor (MICS-2714, Amphenol, Wallingford, Connecticut, USA) sensor was estimated from the manual of the sensors [48] as $log_{10}\left(C_{NOx}\right)≅0.494\times \log_{10}\left(\frac{Rs}{R0}\right)-1.76,$ where $C_{NOx}$ is the concentration of NOx, $R_{0}$ is the initial resistance of the sensor (assumed to be in a clean environment), and $R_{S}$ is the measured value. However, excess NOx was used instead of the absolute value of the NOx concentration to simplify the process. It was noticed that the concentration of NOx should not be higher than 0.1 ppm. According to the datasheet, if $\frac{R_{S}}{R_{0}}>35,$ then the system warns about the existence of NOx.

For the multifunctional sensor (BME680, Bosch Sensortec, Reutlingen, Germany), only the raw data are collected. The raw data include relative humidity, temperature, ambient pressure, and an internal resistance value regarding air quality_._ Here, the MCU collects the raw data and sends them to the mobile phones without processing, so that the size and cost of the MCU for fabrication can be reduced.

**Figure 8:**
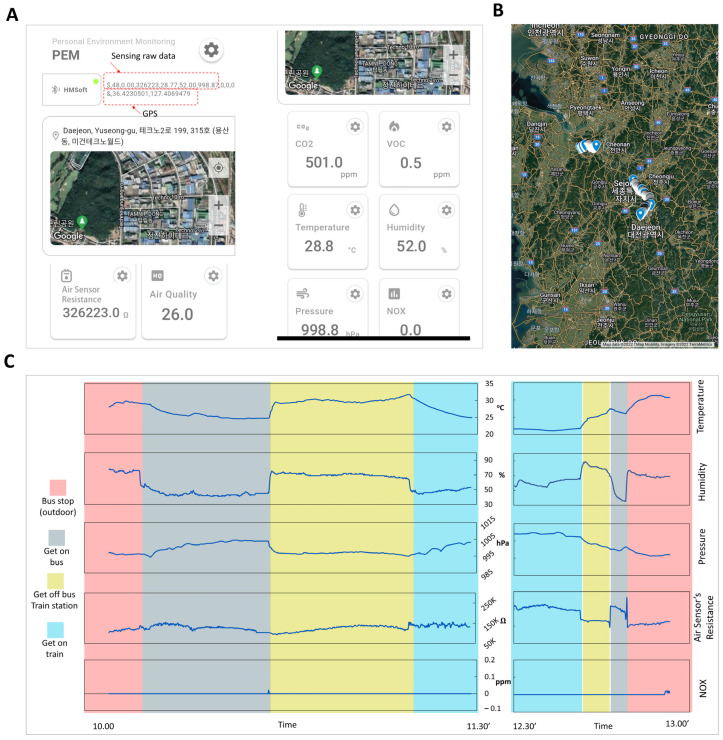
Mobile application and on-the-go monitoring of air quality. (**A**) Mobile application made for monitoring (**B**) and (**C**) measuring results with locations during travel (The Hangeul characters shows the locations in native language).

The mobile phone acts as an intermediary between the personal device and the server; it receives the data from the monitoring devices, communicates with the server, and interacts with the user. A mobile application was specifically developed and investigated for these purposes (Figure 8). Figure 8A shows the interface of the application, wherein the raw data received from the device are shown. The mobile application also includes a location based on the global positioning system (GPS). All the data are packaged and sent to the server for calculations to substantiate the information further.

On the server, an external library from the manufacturer (BSEC—Bosch Sensortec, Germany) [49] is used to interpret air quality from the raw data of BME680. In this way, complete environmental information can be achieved. The calculated data (equivalent CO_2_ concentration (eCO_2_), equivalent VOC concentration (eVOC), and air quality (indoor air quality index (IAQ)) are sent back to the mobile application for personal use (Figure 8A). As per the datasheet of the BME sensor, the IAQ value ranges from 0 to more than 351. The lower the IAQ value, the better the air quality. If the IAQ is under 50, the air quality is very good. The calculation time and data transfer from the server to the mobile phones are given in milliseconds; therefore, the delays are negligible, and the data are considered to be transferred in real time.

To demonstrate the device’s work, the information, including the locations and raw monitored data from the fabricated device during travel of around 100 km for about 3 h, were acquired from the mobile app (Figure 8B,C). The changes in the air quality of the environment were observable. For example, air quality was controlled well inside vehicles, the temperature was about 24 °C, and humidity was about 50%. Moreover, NOx concentration in the environment is within the acceptable range (<0.1 ppm). As noted, the resistance of the air sensor is higher for better air quality, and the air quality variations throughout the trip can be observed.

**Figure 9:**
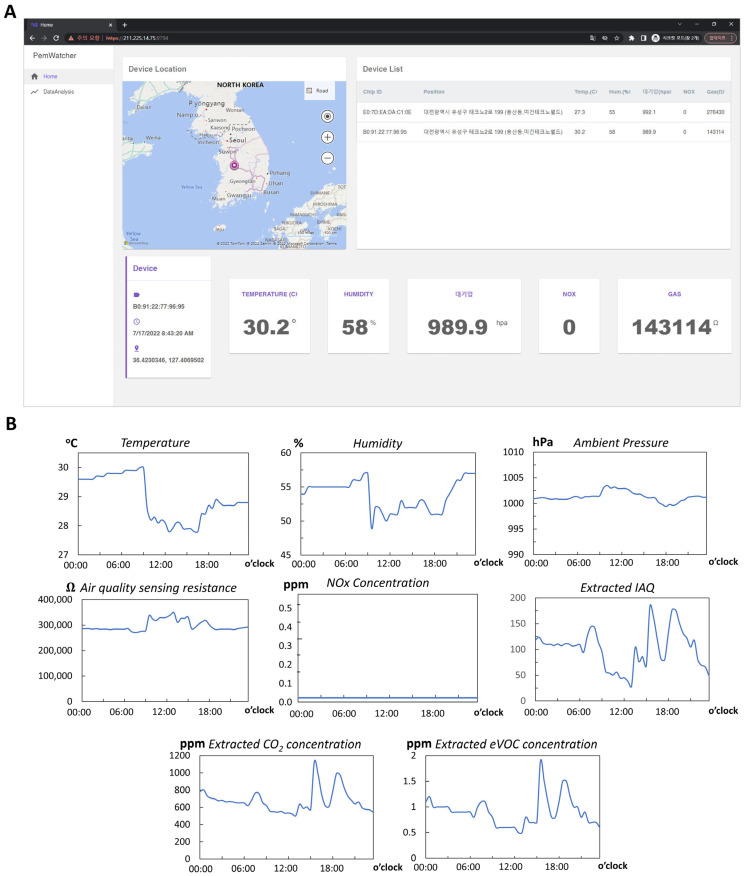
Server for remote monitoring and data analysis. (**A**) Real-time monitoring of air conditions and (**B**) data history for analysis extracted from the server software (The Hangeul characters shows the locations in native language).

The monitored data could be used for further analysis. For instance, the server could show the environmental conditions based on the provided and logged information. Moreover, the fabricated devices could also be used to monitor the air quality at a fixed location over time. Figure 9B shows the change in air quality in an office for one day. In this implementation, the device can be used to determine whether air quality is well regulated during office hours (9:00–18:00) or detect potential human health risk factors.

**Figure 10:**
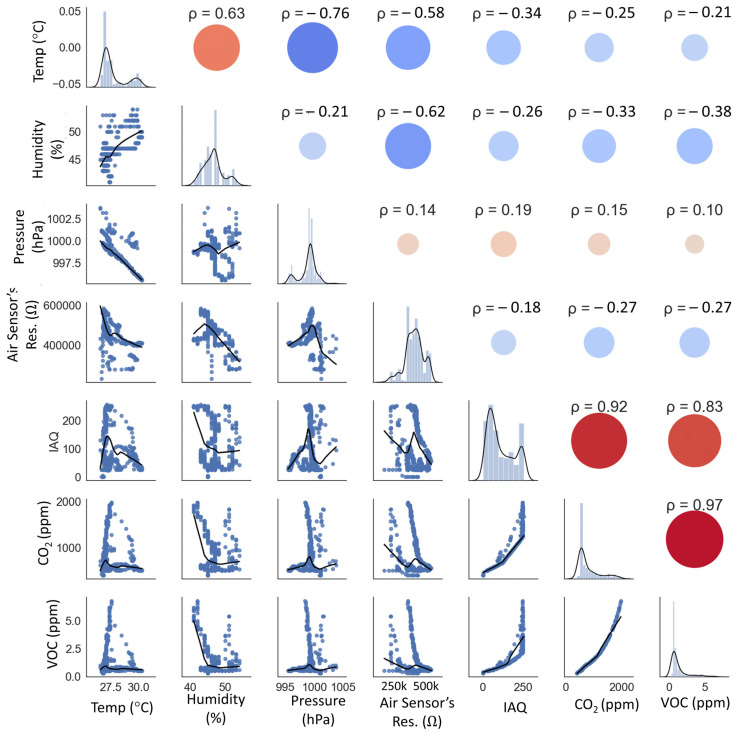
Correlation analysis of the measured values with relationship scatter plots (blue dots), distribution plots (histogram bar plots), and Pearson coefficients (ρ). A larger bubble size shows a stronger correlation.

Further analysis could also be performed on the server using the collected data. For example, the working function of the sensors and the relationship between environmental factors could be investigated (Figure 10), which shows the correlations with the factors’ Pearson coefficients (ρ). According to the factor analysis in Figure 10, the sensor’s sensing resistance value is not affected by ambient pressure. The IAQ value is directly related to the concentration of CO_2_ and VOC. The IAQ, eCO_2_, and eVOC can be computed reciprocally using quasi-linear functions.

## 5. Conclusions

This study proposed the use of R2R printing to develop a hybrid device for a personal environmental monitoring system. Fabricating these devices involved printing multilayer circuits and assembling them with solid-state devices and sensors. Design rules were suggested to enhance fabrication processes and working performance. Passive compensation was used when converting the design into a printable format (passive registration control), and active compensation was used during printing the layers with an in-house R2R screen-printing system.

A personal environmental monitoring system was developed and demonstrated using fabricated devices with a mobile application and a cloud server. The successful implementation of the system paves the way for further development of the system with additional tools, such as big data and artificial intelligence, for practical uses. Other IoT systems can be mass-manufactured and analyzed using similar fabrication concepts and methodologies.

## Figures and Tables

**Table 1:** Design rules used for circuit design.

Parameter	Design Rule	Demonstration
Conductive trace	Linewidth ≥ 200 μm Power line linewidth ≥ 500 μm Line clearance ≥ 500 μm	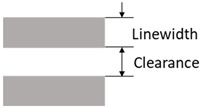
Via holes	Printed insulating hole’s diameter $R_{hole}\geq$ 400 μm Hole cover diameter $R_{cov}\geq$ 600 μm In general: $R_{cov}\geq R_{hole}+200\mu m$	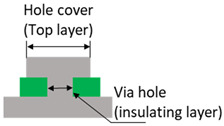
Passive devices	Device size: 1608 (metric) and above Component clearance: ≥ 800 μm	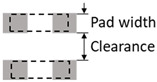
Land patterns of active devices	Pad width ≥ 300 μm Pad clearance ≥ 200 μm	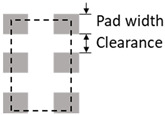
Connectors	Printed FPC connectors at the edges of the circuit (for infrequently used connections)	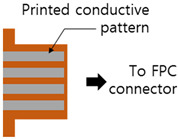

**Table 2:** Scale factors of the original design for passive compensation.

	Layer 1 (Bottom Layer)	Layer 2 (Insulating Layer)	Layer 3 (Top Layer)	Layer 4 (Solder Resist)
**MD**	1.00004	1.00222	1.00237	1.00202
**CD**	0.99476	0.99344	0.99295	0.99213

**Table 3:** Screen mask, materials, and printing conditions.

	Layer 1 (Bottom Layer)	Layer 2 (Insulating)	Layer 3 (Top Layer)	Layer 4 (Solder Resist)
**Printing material**	Silver paste (T40, FP Co., Ltd.)	SFR-300PIY (Seoul Chemical Research Laboratory Co., Ltd.)	Silver paste (T40, FP Co., Ltd.)	SFR-300PIY (Seoul Chemical Research Laboratory Co. Ltd.)
**Screen mesh-count**	400	200	325	325
	Screen mask size: 650 mm × 650 mm			
**Printing condition**	Force: 13 kgf	Force: 11 kgf	Force: 13 kgf	Force: 13 kgf
	The gap between mesh and substrate: 3 mm Squeeze’s speed: 100 mm/s			
**Sintering/annealing**	150 °C–10 min			

## Data Availability

Data presented in this study are available on request from the corresponding author.

## References

[1] Kampa M., Castanas E. (2008). Human health effects of air pollution. Environ. Pollut..

[2] Chen W.Y., Yen C.-C., Xue S., Wang H., Stanciu L.A. (2019). Surface Functionalization of Layered Molybdenum Disulfide for the Selective Detection of Volatile Organic Compounds at Room Temperature. ACS Appl. Mater. Interfaces.

[3] Lv Z., Hu B., Lv H. (2019). Infrastructure Monitoring and Operation for Smart Cities Based on IoT System. IEEE Trans. Ind. Inform..

[4] Malche T., Maheshwary P., Kumar R. (2019). Environmental Monitoring System for Smart City Based on Secure Internet of Things (IoT) Architecture. Wirel. Pers. Commun..

[5] Ullo S.L., Sinha G.R. (2020). Advances in Smart Environment Monitoring Systems Using IoT and Sensors. Sensors.

[6] Hajjaji Y., Boulila W., Farah I.R., Romdhani I., Hussain A. (2020). Big data and IoT-based applications in smart environments: A systematic review. Comput. Sci. Rev..

[7] Castrejon-Pita J.R., Baxter W.R.S., Morgan J., Temple S., Martin G.D., Hutchings I.M. (2013). Future, opportunities and challenges of inkjet technologies. At. Sprays.

[8] Bonnassieux Y., Brabec C.J., Cao Y., Carmichael T.B., Chabinyc M.L., Cheng K.-T., Cho G., Chung A., Cobb C.L., Distler A. (2021). The 2021 flexible and printed electronics roadmap. Flex. Print. Electron..

[9] Shih B., Christianson C., Gillespie K., Lee S., Mayeda J., Huo Z., Tolley M.T. (2019). Design Considerations for 3D Printed, Soft, Multimaterial Resistive Sensors for Soft Robotics. Front. Robot. AI.

[10] Chang T., Wojcik C., Su Y., Rogers J.A., Lee T.H., Fan J.A. (2017). Characterization of stretchable serpentine microwave devices for wearable electronics. 2017 IEEE MTT-S International Microwave Symposium (IMS).

[11] Xie Z., Ji B., Huo Q. (2018). Mechanics Design of Stretchable Near Field Communication Antenna With Serpentine Wires. J. Appl. Mech..

[12] Liu G., Tan Q., Kou H., Zhang L., Wang J., Lv W., Dong H., Xiong J. (2018). A Flexible Temperature Sensor Based on Reduced Graphene Oxide for Robot Skin Used in Internet of Things. Sensors.

[13] Gao M., Li L., Song Y. (2017). Inkjet printing wearable electronic devices. J. Mater. Chem. C.

[14] Zavanelli N., Yeo W.-H. (2021). Advances in Screen Printing of Conductive Nanomaterials for Stretchable Electronics. ACS Omega.

[15] Liu C., Huang N., Xu F., Tong J., Chen Z., Gui X., Fu Y., Lao C. (2018). 3D Printing Technologies for Flexible Tactile Sensors toward Wearable Electronics and Electronic Skin. Polymers.

[16] Fernandes D.F., Majidi C., Tavakoli M. (2019). Digitally printed stretchable electronics: A review. J. Mater. Chem. C.

[17] Nagamine K., Nomura A., Ichimura Y., Izawa R., Sasaki S., Furusawa H., Matsui H., Tokito S. (2020). Printed Organic Transistor-based Biosensors for Non-invasive Sweat Analysis. Anal. Sci..

[18] Iqbal S.M.A., Mahgoub I., Du E., Leavitt M.A., Asghar W. (2021). Advances in healthcare wearable devices. NPJ Flex. Electron..

[19] Liao Y., Zhang R., Qian J. (2019). Printed electronics based on inorganic conductive nanomaterials and their applications in intelligent food packaging. RSC Adv..

[20] Wang Y.-F., Sekine T., Takeda Y., Yokosawa K., Matsui H., Kumaki D., Shiba T., Nishikawa T., Tokito S. (2020). Fully Printed PEDOT:PSS-based Temperature Sensor with High Humidity Stability for Wireless Healthcare Monitoring. Sci. Rep..

[21] Dong Y., Bao C., Kim W.S. (2018). Sustainable Additive Manufacturing of Printed Circuit Boards. Joule.

[22] Hakola L., Jansson E., Futsch R., Happonen T., Thenot V., Depres G., Rougier A., Smolander M. (2021). Sustainable roll-to-roll manufactured multi-layer smart label. Int. J. Adv. Manuf. Technol..

[23] Jo M., Kim S., Cho G., Lee T.-M., Lee J., Lee C. (2022). Achieving specified geometric quality in a fully printed flexible functional layer using process parameters in roll-to-roll printed electronics. Flex. Print. Electron..

[24] Gafurov A.N., Phung T.H., Ryu B.-H., Kim I., Lee T.-M. (2022). AI-Aided Printed Line Smearing Analysis of the Roll-to-Roll Screen Printing Process for Printed Electronics. Int. J. Precis. Eng. Manuf. Technol..

[25] Kim K., Kim J., Kim B., Ko S. (2018). Fabrication of Microfluidic Structure Based Biosensor Using Roll-to-Roll Gravure Printing. Int. J. Precis. Eng. Manuf. Technol..

[26] Cao X.B., Hoang L.P., Kim C.N.T., Vu T.T. (2023). Laser ablation on coated metal gravures for roll-to-roll printed electronics. Opt. Commun..

[27] Shrestha S., Parajuli S., Park J., Yang H., Cho T.-Y., Eom J.-H., Cho S.-K., Lim J., Cho G., Jung Y. (2023). Improving Stability of Roll-to-Roll (R2R) Gravure-Printed Carbon Nanotube-Based Thin Film Transistors via R2R Plasma-Enhanced Chemical Vapor-Deposited Silicon Nitride. Nanomaterials.

[28] Tiara A.M., Moon H., Cho G., Lee J. (2022). Fully roll-to-roll gravure printed electronics: Challenges and the way to integrating logic gates. Jpn. J. Appl. Phys..

[29] Phung T.H., Jeong J., Gafurov A.N., Kim I., Kim S.Y., Chung H.-J., Kim Y., Kim H.-J., Kim K.M., Lee T.-M. (2021). Hybrid fabrication of LED matrix display on multilayer flexible printed circuit board. Flex. Print. Electron..

[30] Liu Q., Tian B., Liang J., Wu W. (2021). Recent advances in printed flexible heaters for portable and wearable thermal management. Mater. Horizons.

[31] Maskey B.B., Lee J., Majima Y., Kim J., Lee J., Bahk G., Koirala G.R., Cho G., Sun J., Shrestha K. (2019). A Smart Food Label Utilizing Roll-to-Roll Gravure Printed NFC Antenna and Thermistor to Replace Existing “Use-By” Date System. IEEE Sensors J..

[32] Bariya M., Shahpar Z., Park H., Sun J., Jung Y., Gao W., Nyein H.Y.Y., Liaw T.S., Tai L.-C., Ngo Q.P. (2018). Roll-to-Roll Gravure Printed Electrochemical Sensors for Wearable and Medical Devices. ACS Nano.

[33] Kim Y.Y., Yang T.-Y., Suhonen R., Kemppainen A., Hwang K., Jeon N.J., Seo J. (2020). Roll-to-roll gravure-printed flexible perovskite solar cells using eco-friendly antisolvent bathing with wide processing window. Nat. Commun..

[34] Jung Y., Shrestha S., Lim N., Park H., Sun J., Park J., Parajuli S., Shrestha K., Kim S., Cho G. (2022). A Printed Wireless Triangle-Wave Generator via a Smartphone. Adv. Eng. Mater..

[35] Ge L., Ye X., Yu Z., Bin Chen B., Liu C., Guo H., Zhang S., Sassa F., Hayashi K. (2022). A fully inkjet-printed disposable gas sensor matrix with molecularly imprinted gas-selective materials. NPJ Flex. Electron..

[36] Khan Y., Thielens A., Muin S., Ting J., Baumbauer C., Arias A.C. (2019). A New Frontier of Printed Electronics: Flexible Hybrid Electronics. Adv. Mater..

[37] Luoma E., Välimäki M., Ollila J., Heikkinen K., Immonen K. (2022). Bio-Based Polymeric Substrates for Printed Hybrid Electronics. Polymers.

[38] Ahmadi Z., Lee S., Patel A., Unocic R.R., Shamsaei N., Mahjouri-Samani M. (2022). Dry Printing and Additive Nanomanufacturing of Flexible Hybrid Electronics and Sensors. Adv. Mater. Interfaces.

[39] Ma Y., Zhang Y., Cai S., Han Z., Liu X., Wang F., Cao Y., Wang Z., Li H., Chen Y. (2019). Flexible Hybrid Electronics for Digital Healthcare. Adv. Mater..

[40] Kasi V., Zareei A., Gopalakrishnan S., Alcaraz A.M., Joshi S., Arfaei B., Rahimi R. (2023). Flexible Hybrid Electronics via Near-Infrared Radiation-Assisted Soldering of Surface Mount Devices on Screen Printed Circuits. Adv. Electron. Mater..

[41] Rogers J.A., Chen X., Feng X. (2020). Flexible Hybrid Electronics. Adv. Mater..

[42] Shiwaku R., Matsui H., Nagamine K., Uematsu M., Mano T., Maruyama Y., Nomura A., Tsuchiya K., Hayasaka K., Takeda Y. (2018). A Printed Organic Amplification System for Wearable Potentiometric Electrochemical Sensors. Sci. Rep..

[43] Burgués J., Marco S. (2018). Low Power Operation of Temperature-Modulated Metal Oxide Semiconductor Gas Sensors. Sensors.

[44] Phung T.H., Gafurov A.N., Kim I., Kim S.Y., Kim K.M., Lee T.-M. (2021). IoT device fabrication using roll-to-roll printing process. Sci. Rep..

[45] Gafurov A.N., Jeong J., Park P., Kim I., Phung T.H., Kim H.-C., Kang D., Oh D., Lee T.-M. (2021). Registration error analysis and compensation of roll-to-roll screen printing system for flexible electronics. Flex. Print. Electron..

[46] Son M.-J., Kim H., Maeng S., Lee T.-M., Lee H.-J., Kim I. (2021). Improvement of electrical and mechanical properties of In-48Sn solder bumps for flexible LED signage using Cu-Ag nanoparticles. Flex. Print. Electron..

[47] Schirmer J., Klauß M., Reichenberger M., Hümmer M., Neermann S., Franke J. (2021). Evaluation of detatchable board-to-board interconnects on screen printed electronic structures. Flex. Print. Electron..

[48] Amphenol (2014). MiCS-2714 NO2 Sensor Datasheet Rev 6.

[49] Bosch Sensortec (2022). BME680 Datasheet v1.8.

